# Weight loss on stimulant medication: how does it affect body composition and bone metabolism? – A prospective longitudinal study

**DOI:** 10.1186/1687-9856-2012-30

**Published:** 2012-12-05

**Authors:** Alison Poulton, Julie Briody, Thomas McCorquodale, Elaine Melzer, Markus Herrmann, Louise A Baur, Gustavo Duque

**Affiliations:** 1 Department of Paediatrics, Sydney Medical School Nepean, The University of Sydney, Penrith, NSW, Australia; 2 Department of Nuclear Medicine, The Children’s Hospital at Westmead, Sydney, NSW, Australia; 3 Ageing Bone Research Program, Sydney Medical School Nepean, The University of Sydney, Penrith, NSW, Australia; 4 Central Laboratory of Clinical Pathology, Central Hospital of Bolzano, Bolzano, Italy; 5 University of Sydney Discipline of Paediatrics and Child Health, The Children’s Hospital at Westmead, Sydney, NSW, Australia

**Keywords:** Body composition, Attention deficit hyperactivity disorder, Stimulant medication, Bone biochemistry, Weight loss

## Abstract

**Objective:**

Children treated with stimulant medication for attention deficit hyperactivity disorder (ADHD) often lose weight. It is important to understand the implications of this during growth. This prospective study was designed to quantify the changes in body composition and markers of bone metabolism on starting treatment.

**Methods:**

34 children (29 boys) aged 4.7 to 9.1 years newly diagnosed with ADHD were treated with dexamphetamine or methylphenidate, titrating the dose to optimise the therapeutic response. Medication was continued for as long as clinically indicated. Body composition and bone density (dual-energy X-ray absorptiometry) were measured at baseline, 6 months and 3 years; changes were analysed in Z-scores based on data from 241 healthy, local children. Markers of bone turnover were measured at baseline, 3 months and 3 years.

**Results:**

Fat loss of 1.4±0.96kg (total fat 5.7±3.6 to 4.3±3.1kg, p<0.001) occurred in the first 6 months. There were significant reductions over 3 years in the sex and height corrected Z-scores for lean tissue, bone mineral content, bone mineral density and ratio of central to total fat (−0.84±0.86, p=0.003; -0.55±0.31, p<0.0001; -0.41±0.28, p<0.0001 and −0.55±0.62, p=0.006 respectively). Propeptide of type I collagen indicated a significant reduction in bone turnover after 3 months (564±202 to 458±96ng/ml, p=0.019), which was fully recovered after 3 years (619±276ng/ml).

**Conclusions:**

Stimulant medication was associated with early fat loss and reduced bone turnover. Lean tissue including bone increased more slowly over 3 years of continuous treatment than would be expected for growth in height. There was long-term improvement in the proportion of central fat for height. This study shows that relatively minor reductions in weight on stimulant medication can be associated with long-term changes in body composition. Further study is required to determine the effects of these changes on adult health.

## Background

The stimulant medications dexamphetamine and methylphenidate are used for the treatment of children with attention deficit hyperactivity disorder (ADHD) because they reduce the level of hyperactivity and enhance the attention span. Weight loss and reduced height velocity are well-recognized collateral effects [1-3], particularly in the first 6 months of treatment. In a previous study we demonstrated that the growth rates for height and weight progressively normalized over 2–3 years on treatment, but the period of slower growth was associated with long-term reductions in the age and sex corrected Z-scores for height and weight [4]. These observations raise the following important questions: what is the nature of the tissue being lost and what is the effect on bone development?

The aims of this prospective study were to monitor growth in children with ADHD starting treatment with stimulant medication and to look for changes in body composition and markers of bone metabolism. The hypotheses informing the study design were (1) that acute changes in hormones and bone biomarkers during weight loss would be observed in the first 3 months of treatment; (2) that changes in body composition would be detectable after 6 months and (3) a new steady state of normal growth on stimulant medication would be reached after 3 years of continuous treatment.

## Methods

### Design and setting

This was a clinic based, prospective, cohort study with up to three years follow up. Recruitment was mainly from a single pediatric private practice in western Sydney; two other practices each contributed one patient. Recruitment began in July 2003 and continued until April 2008. Ethical approval was granted by the Human Research Ethics Committee of Wentworth Area Health Service in western Sydney (02/013).

### Study participants

We invited parents of consecutive children aged <9 years who were newly diagnosed with ADHD and had a clinical indication for starting treatment with stimulant medication to enroll their children in the study. The diagnosis of ADHD was based on clinical interview with additional information on functioning at school or preschool. All children met the diagnostic criteria of the American Psychiatric Association (DSM-IV) [5]. Children with a history of previous treatment with psychotropic medication or with medical conditions likely to impact on growth were excluded. Informed consent was given by the parents of all participants; all participating children assented to the study.

### Treatment protocol

All children were initially trialed on immediate release methylphenidate or dexamphetamine; some children were subsequently prescribed extended release formulations. Treatment allocation was based on cost, with dexamphetamine being the preferred medication until methylphenidate also became subsidised. The dose was titrated to give the maximum therapeutic benefit at the lowest possible dose of medication, in line with recommended practice parameters [6]. Children who were stable and functioning well on stimulant medication were reviewed every 6 months and the dose adjusted when clinically indicated. This decision was based on parental reports of the child’s functioning, the child’s opinion, school reports and other documentation from the school, including the IOWA Conner’s rating scale [7]. If at any stage there was no clear advantage to stimulant medication and the child was functioning adequately, medication was ceased and the child’s functioning reassessed off medication. No further data were collected on children who had ceased medication.

### Data collection

We measured height to the nearest 1mm using a wall mounted stadiometer and weight to the nearest 0.1kg using electronic scales. All measurements were made without shoes or outdoor clothing. Measurements were taken at every clinic visit and without prior reference to any previous measurements.

Body mass index (BMI) was calculated as the weight in kg divided by the square of the height in meters and the height, weight and BMI were corrected for age and sex by conversion to Z-scores based on Centers for Disease Control and Prevention (CDC) reference data [8].

Dual-energy X-ray absoptiometry (DXA) scans of the Total Body and lumbar spine were performed using a GE-Lunar Prodigy (GE Lunar Corp, Madison, WI). Daily quality assurance and quality control (spine phantom in water bath) were performed.We measured total body (including head) and body subregions (arms, legs, trunk) lean tissue and fat masses, bone mineral content (BMC), bone mineral density (BMD) and fat distribution (ratio of central to total fat; fat C/T). For estimation of the central (truncal) fat, the trunk region (which includes the pelvis) was delineated by a single assessor using standard manufacturer analysis guidelines. The assessor (JB) has shown good inter-rater reliability with a colleague [9]. Lumbar spine (L2-L4), BMD and BMC were also determined. Raw DXA values were converted into Z-scores using DXA control data.

DXA control data measured using a Lunar DPX (GE Lunar Corp, Madison, WI) were available from 241 healthy children (105 boys), aged 4.2-12.0 years (mean 8.54±1.93 years). Parents of these children had consented for them to have a single DXA to provide reference data for research purposes. Although there was no direct comparison of the Lunar DPX and the Lunar Prodigy used for the subjects, our data from 97 children comparing another Lunar Prodigy with the Lunar DPX showed no significant differences in BMC or BMD. There were small differences in lean tissue mass (mean 28.6 vs 29 kg), and fat (mean 11.4 vs 12.3 kg) but the results were all highly correlated (all r^2^ > 0.98).

Blood samples were collected unmedicated after an 8 hour overnight fast. The following bone turnover markers and calciotropic hormones were measured: osteocalcin, 25-hydroxy vitamin D, parathyroid hormone (PTH), amino-terminal propeptide of type I collagen (P1NP) and C-telopeptides (CTX). In addition, appetite regulating hormones (leptin and ghrelin) and the growth hormone dependent growth factors insulin-like growth factor 1 (IGF-1) and insulin-like growth factor binding protein 3 (IGFBP-3), together with fasting insulin, glucose albumin, pre-albumin, ferritin and transferrin were measured to look for changes in growth and appetite regulation and for biochemical evidence of undernutrition. Vitamin D and osteocalcin were analysed by chemiluminescence immunoassays on a Diasorin Liaisonanalyzer (Diasorin, Italy). CTX and P1NP were measured with commercial electrochemiluminescence immunoassays (Modular E170, Roche Diagnostics, Australia). Albumin was analysed using an automated colorimetric assay using a Vitros Fusion analyser (Ortho Clinical Diagnostics, Australia). Pre-albumin used an immunoturbid metric assay on an Integra (Roche Diagnostics, Australia). Ferritin was assayed using an immunometric assay on a Vitros ECI (Ortho Clinical Diagnostics, Australia). Iron deficiency was defined as ferritin <20 ug/L. Transferrin used the TRNF method, which is a quantitative turbid metric assay (Dimension Xpand, Siemens Medical Solutions Diagnostics Pty Ltd, Sydney, Australia). Glucose was analysed using routine laboratory procedures. Leptin and total ghrelin levels were analysed using radioimmunoassay (Millipore, MA) and insulin by chemiluminescence (IMMULITE 2000®, Siemens Medical Solutions Diagnostics Pty Ltd, Sydney, Australia). PTH, IGF-I and IGFBP-3 were measured by ELISA using the IMMULITE 1000® analyser (Siemens Medical Solutions Diagnostics Pty Ltd, Sydney, Australia).

### Sample size calculations

These were based on the growth data from our previous study [4]. The estimated sample sizes required to detect a change in height Z-score with 90% power at 5% two sided significance were 16 and 11 after 6 months and 3 years respectively; for weight Z-score these were 8 and 13 respectively. Therefore we aimed to recruit 30 children, to allow for 50% attrition at 3 years.

### Outcome measures

#### Growth

Growth was analyzed as changes in height, weight and BMI Z-scores.

#### Body composition

Body composition was analysed as follows:

1. Longitudinal changes in lean tissue, fat, BMC, BMD and fat distribution (fat C/T).

2. Cross sectional comparison of lean tissue, fat, BMC, BMD and fat C/T at each timepoint with the DXA control data, controlling for age, sex and height.

3. Longitudinal changes in lean tissue, fat, BMC, BMD and fat C/T at 6 months and 3 years in relation to growth in height using sex and height corrected Z-scores based on the DXA control data. Height was used instead of age as we considered this to be the more clinically relevant measure if height was not changing proportionally with age due to stimulant associated changes in the height velocity.

### Statistical analysis

We used paired t-tests for longitudinal data analysis and independent sample t-tests for cross sectional comparison. The DXA data from the subjects were compared to the DXA control data using general linear modeling with age, sex and height as independent variables. Correlations between variables used the Pearson correlation. All analyses were 2-tailed and statistical significance was taken as p<0.05.

## Results

### Participant characteristics

**Figure 1:**
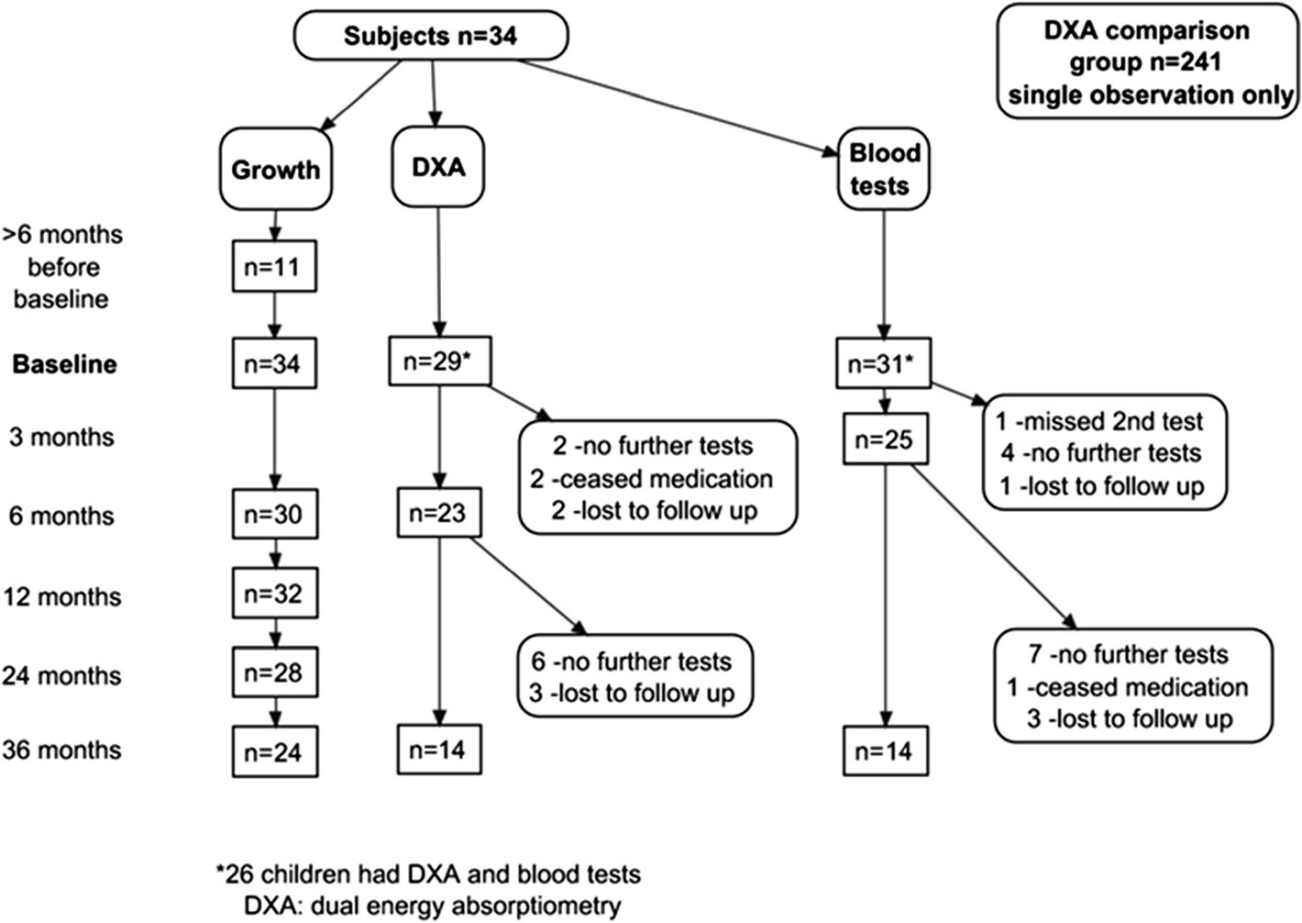
Number of subjects and attrition in each part of the study.

Thirty-four children aged 4.7 to 9.1 years (mean 7.3±1.3 years, 29 boys) were recruited. Of these, 29 had baseline DXA scans and 31 had baseline blood tests (Figure 1). All met a DSM-IV diagnosis of ADHD and the majority (78%) had hyperactivity-impulsivity as well as inattention (combined type). The majority of children were treated with methylphenidate: at 6 months there were 23 on methylphenidate (mean 24.3±6.2 mg/day; 0.91±0.19 mg/kg/day) and 7 on dexamphetamine (mean 11.1±2.8 mg/day; 0.42±0.08 mg/kg/day). The attrition rate for investigations was 53% (18/34) at 3 years. Eight of these children were still being treated with stimulant medication and had growth data available; the remaining 10 (29% of the cohort) had either ceased medication (n=3), had moved away (n=1) or were otherwise lost to follow up (n=6). There were no significant differences in age or growth parameters between those who did and those who did not complete their investigations, therefore the growth data of all the children who remained on treatment have been included in the tables and figures.

### Growth rates

**Figure 2:**
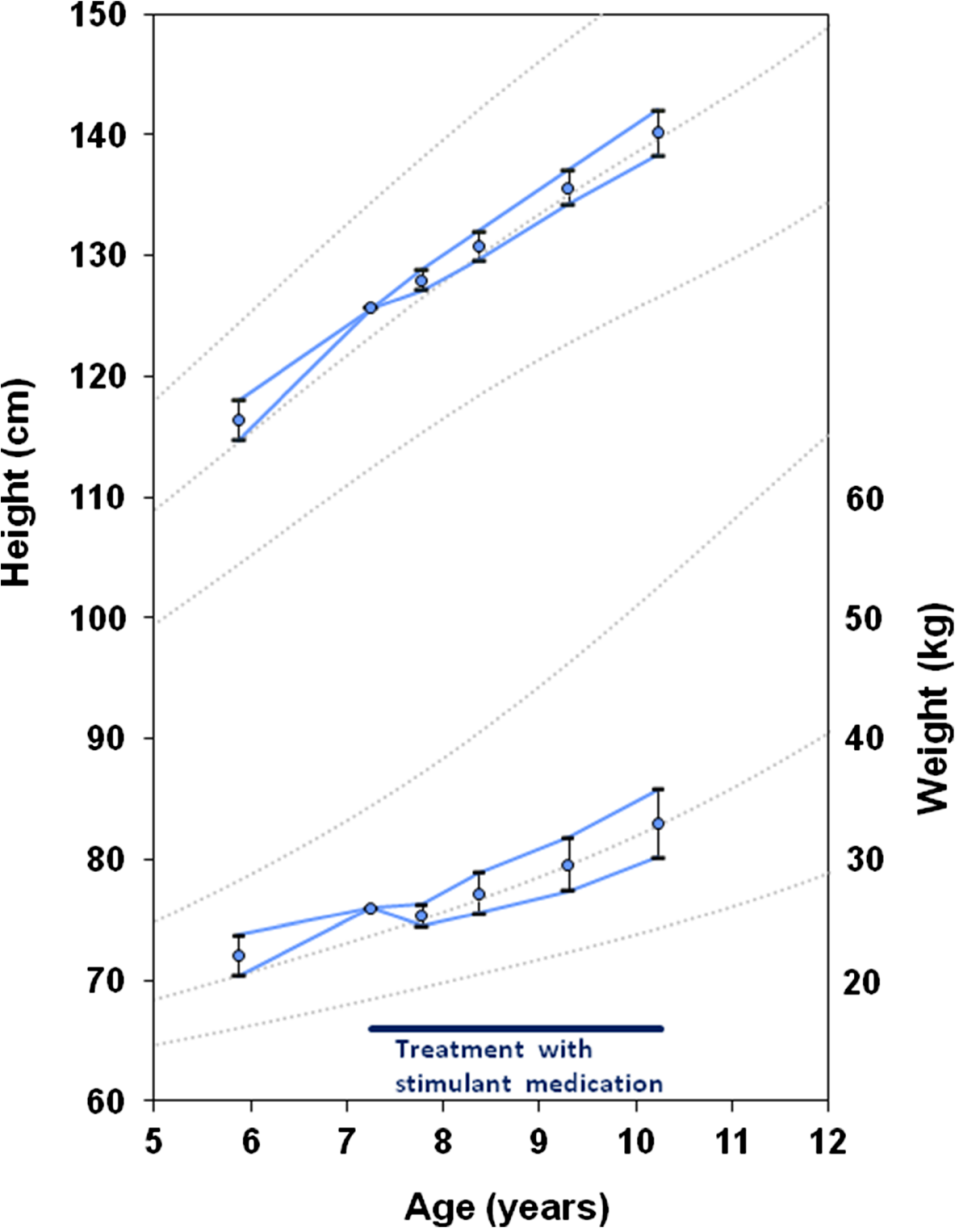
**Growth chart showing the average height and weight calculated from the changes in Z-scores.** The error bars denote the standard deviation of the change in Z-scores going forwards and backwards from the baseline data, standardised for the average baseline age. The data show initial weight loss with simultaneous slowing of the height velocity on starting stimulant medication. Reference data: Centres for Disease Control and Prevention (CDC) 2000; mean ± 2SD.

At baseline the mean Z-scores for height, weight and BMI were 0.49 ± 0.99, 0.62 ± 0.97 and 0.52 ± 1.02 respectively (Table 1). Over 3 years there were significant reductions from baseline in the Z-scores for height, weight and BMI (all p<0.001). This was predominantly due to slower growth in the first 6 months (Table 2); the growth rate showed normalization with time (p<0.01 and p<0.001 for the difference in the rate of change from 0–6 months and from 6 months to 3 years for height and weight Z-scores respectively). The growth pattern is illustrated in Figure 2.

**Table 1:** Growth data of the subjects compared to the controls

	**All subjects**		**All subjects**		**All subjects**		**DXA Controls**	
	**Baseline n=34**		**6 months n=30**		**36 months n=24**		**n=241**	
	**Mean ± sd**	**Range**	**Mean ± sd**	**Range**	**Mean ± sd**	**Range**	**Mean ± sd**	**Range**
Age (years)	7.27 ± 1.30***	4.71-9.12	7.99 ± 1.21*	5.33-9.64	10.49 ± 1.22***	7.87-12.21	8.54 ± 1.93	4.02-11.99
Height (cm)	125.5 ± 9.1*	110.3-142.2	128.6 ± 9.2	112.6-145.6	140.6 ± 9.0***	125.5-156.0	130.5 ± 12.7	94.5-161.6
Weight (kg)	27.0 ± 6.1	19.1-42.1	26.8 ± 5.9	19.3-42.2	34.4 ± 8.4**	25.0-56.0	29.0 ± 8.2	13.1-60.4
BMI (kg/m^2^)	17.0 ± 2.3	13.7-23.7	16.1 ± 2.3	12.9-23.0	17.3 ± 3.1	13.9-26.5	16.7 ± 2.2	13.3-25.6
Height Z-score	0.49 ± 0.99**	−1.08-3.02	0.22 ± 0.95	−1.19-2.76	−0.07 ± 0.81	−1.18-1.67	−0.06 ± 0.97	−2.24-2.07
Weight Z-score	0.62 ± 0.97***	−1.60-2.95	0.04 ± 1.04	−1.86-2.63	−0.12 ± 0.87	−1.48-1.58	−0.05 ± 0.92	−2.25-2.23
BMI Z-score	0.52 ± 1.02**	−1.88-2.31	−0.17 ± 1.21	−2.92-2.04	−0.16 ± 1.11	−2.45-1.97	0.03 ± 0.88	−2.11-2.09

*p<0.05; **p<0.01; ***p<0.001 compared to DXA controls (independent samples *t*-test).

DXA: Dual-energy X-ray absorptiometry.

**Table 2:** Growth rates of the subjects over different time periods on medication

**Time period (months: mean ± SD)**	**Pre-treatment**	**0-6 months**	**6-12 months**	**12-24 months**	**24-36 months**
	**(16.6 ± 9.4)**	**(6.3 ± 1.5)**	**(7.3 ± 2.0)**	**(11.4 ± 2.1)**	**(10.9 ± 2.5)**
	**n=11**	**n=30**	**n=30**	**n=28**	**n=24**
Height velocity (cm/year)	6.5 ± 1.1	4.3 ± 1.9	4.5 ± 1.3	4.7 ± 1.2	5.3 ± 1.2
Weight velocity (kg/year)	2.7 ± 1.8	−1.5 ± 2.8	3.2 ± 3.1	2.9 ± 2.3	4.1 ± 2.3
Change in BMI/year	0.08 ± 0.86	−2.09 ± 1.89	0.81 ± 1.74	0.45 ± 1.05	0.83 ± 0.95
Δ Height Z-score/year	−0.04 ± 0.17	−0.32 ± 0.38***	−0.22 ± 0.21***	−0.11 ± 0.19**	−0.02 ± 0.17
Δ Weight Z-score/year	−0.09 ± 0.36	−1.04 ± 0.74***	0.03 ± 0.63	−0.13 ± 0.27*	0.06 ± 0.29
Δ BMI Z-score/year	−0.08 ± 0.44	−1.29 ± 0.94***	0.26 ± 0.96	−0.10 ± 0.39	0.12 ± 0.43

*p<0.05; **p<0.01; ***p<0.001 for the change in Z-score over the time period using single sample t-tests against a test value of zero.

Statistical analysis was applied to the changes in Z-scores only and used single sample t-tests with a test value of zero.

### Body composition

Baseline DXA data were available for 29 children, repeated at 6 months (median 6.8 months, mean 7.3±1.8 months) in 24 (19 boys) and at 3 years (median 2.9 years, mean 2.7±0.6 years) in 14 (12 boys).

#### Longitudinal changes in tissue mass

**Figure 3:**
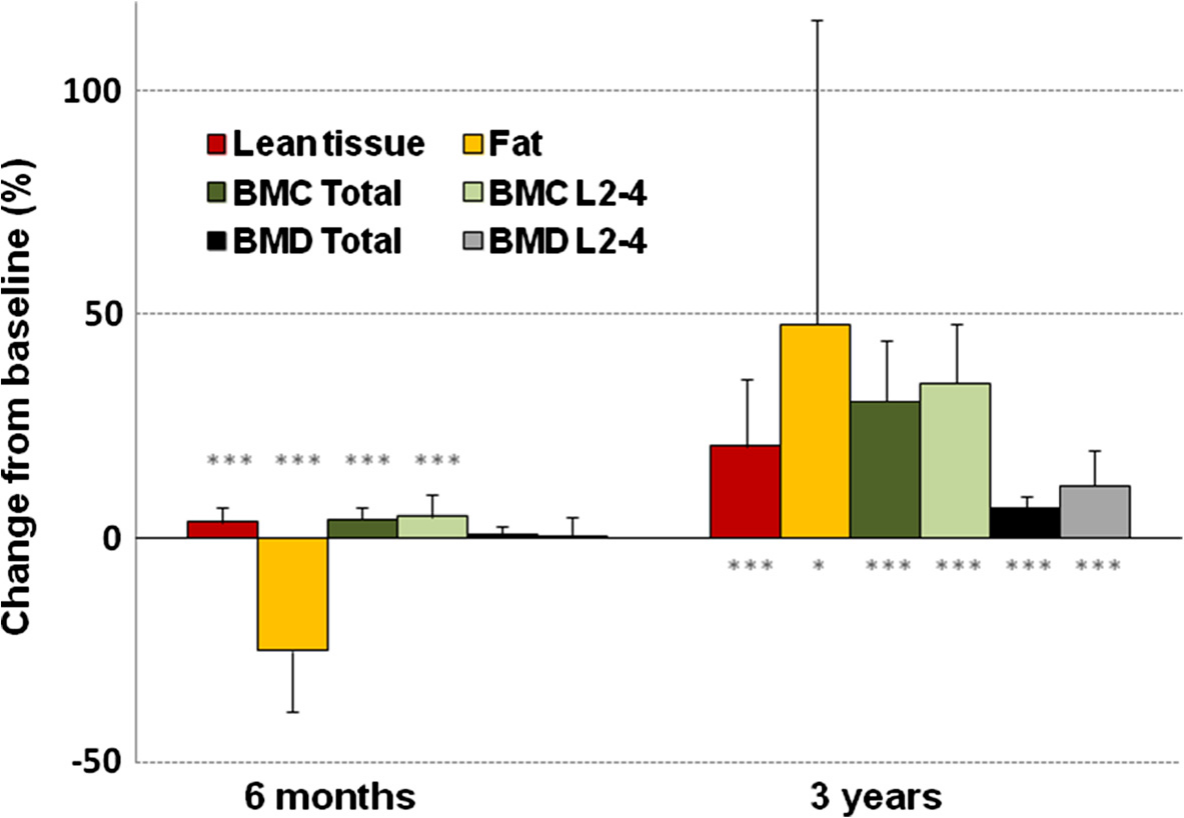
**Percentage change in components of body composition.** BMC: bone mineral content; BMD: bone mineral density 6 months n=23; 3 years n=14 *** p<0.001; ** p<0.01; * p<0.05 from baseline, paired *t*-test. There was a significant reduction in fat mass and significant increases in lean tissue and BMC in the first 6 months. Over 3 years there were significant increases in lean tissue, BMC, BMD and fat.

In the first 6 months the children lost 2.2±3.6% of their total tissue mass (p=0.007), which equated to an average loss of 0.65 ± 1.07 kg of tissue (p=0.009). This was associated with a significant reduction in their fat mass (5.7±3.6 to 4.3±3.1kg, change −1.40 ± 0.96 kg, p<0.001) (Figure 3). The average fat loss was greater than the weight loss because of a significant rise in lean tissue (20.4±3.0 to 21.2±3.1kg, change 0.76±0.64 kg, p<0.001). Total BMC increased significantly (1.02±0.20 to 1.06±0.20kg, change 0.04±0.03 kg, p<0.001). BMD increased significantly in the arms (0.61±0.04 to 0.62±0.04 kg/m^2^, change 0.006±0.012 kg/m^2^, p=0.023) but there was no significant change in the total body, trunk, legs or lumbar spine. Over the period from baseline to 3 years there were significant increases in all tissues.

#### Cross sectional comparison with DXA control data controlling for age, sex and height using general linear modeling

After controlling for age, sex and height the subjects’ BMD and fat C/T were significantly greater than the DXA controls’ at all time points (Table 3). The subjects’ BMC was significantly greater at baseline but there was no significant difference at 6 months or 3 years. The subjects’ lean tissue mass was significantly lower than the DXA controls at 3 years. There was no significant difference at any stage between subjects and DXA controls in their total fat corrected for age, sex and height. Table 3, as well as showing the comparison of the body composition and bone density of the subjects and controls, also indicates the direction of change with time. For example lean tissue increased with age in the controls but decreased in the subjects and after 3 years of treatment the subjects had significantly lower sex, height and age corrected lean tissue than the controls.

**Table 3:** Body composition of the subjects at different times compared to the controls

		**Subjects**		**DXA Controls**	
		**Baseline: n=29 (24 boys)**		**n=241 (105 boys)**	
		**6 months: n=23 (19 boys)**			
		**36 months n=14 (12 boys)**			
		**LS mean**	**Compared to DXA controls**	**LS mean**	**Comparator**
Lean tissue (kg)	Baseline	20.82	p=0.22	21.36	
	6 months	20.79	p=0.14	21.51	
	36 months	20.00	**p=0.007**	21.72	
BMC (kg)	Baseline	1.053	**p=0.04**	1.001	
	6 months	1.052	p=0.13	1.011	
	36 months	1.050	p=0.46	1.024	
BMD (kg/m^2^)	Baseline	0.891	**p<0.0001**	0.849	
	6 months	0.889	**p=0.0002**	0.851	
	36 months	0.889	**p=0.006**	0.854	
Total fat (kg)	Baseline	6.28	p=0.24	5.55	
	6 months	4.84	p=0.25	5.62	
	36 months	5.35	p=0.69	5.71	
Central/total fat	Baseline	0.421	**p<0.0001**	0.349	
	6 months	0.390	**p=0.0009**	0.350	
	36 months	0.383	**p=0.047**	0.352	

LS Mean: Least squares mean and p values compared to DXA controls using general linear model controlling for effects of age, sex and height.

DXA: Dual-energy X-ray absorptiometry.

Statistically significant p-values are given in bold type.

#### Longitudinal changes in sex and height corrected Z-scores

After 6 months the sex and height corrected Z-scores for BMC, BMD, fat and fat C/T all showed significant reductions (0.52±0.80 to 0.34±0.79, p<0.001; 0.54±0.79 to 0.39±0.85, p=0.010; 0.76±1.13 to −0.23±0.97, p<0.000001 and 1.10±0.80 to 0.65±0.88, p=0.003 respectively) but there was no significant change in the lean tissue Z-score for height and sex (−0.05±0.96 to 0.19±1.02, p=0.11). After 3 years the sex and height corrected Z-scores for lean tissue, BMC, BMD and fat C/T all showed significant reductions from their baseline values (0.09±0.89 to −0.74±1.10, p=0.003; 0.57±0.60 to 0.02±0.66, p<0.0001; 0.62±0.67 to 0.21±0.70, p<0.0001; and 1.01±0.80 to 0.46±1.06, p=0.006 respectively). By contrast at 3 years the Z-score for the fat mass for height was not significantly different from baseline (0.41±1.06 to 0.26±1.29, p=0.62).

### Blood tests

Of 31 children who provided baseline blood samples, 25 (81%) provided repeat samples after 3 months and 14 (48%) after 3 years (Table 4). At baseline 10 children (32%) had iron deficiency; these children showed a significant rise in ferritin over 3 years (p=0.04). In the first 3 months there were significant falls in concentrations of leptin and P1NP (4.45±4.03 ng/ml to 2.73±2.03 ng/ml, p<0.010 and 554±176 ng/ml to 458±93ng/ml, p=0.019 respectively) and a significant rise in albumin (45.1 ± 3.7 g/L to 46.5±3.2 g/L, p<0.027). Concentrations of osteocalcin, CTX, IGF-1 and IGFBP-3 increased over 3 years (50.9±21.9 ng/mL to 80.9±22.2 ng/mL, p=0.003; 0.545±0.205 pg/mL to 0.886±0.518 pg/mL, p=0.038; 19.7±7.3 nmol/L to 31.1±18.0 nmol/L, p=0.041; and 4.07±1.08 ug/mL to 5.82±2.75 ug/mL, p=0.034 respectively). There were no significant changes in fasting levels of vitamin D, PTH, prealbumin, ferritin, transferrin, insulin, glucose or ghrelin.

**Table 4:** Biochemistry results at baseline and after 3 months and 3 years

	**Baseline**	**3 months**	**3 years**
	**(n=25-31)**	**(n=17-25)**	**(n=9-15)**
Albumin (g/L)	45.1 ± 3.7	46.5 ± 3.2*	46.1 ± 4.1
Prealbumin (g/L)	0.19 ± 0.04	0.19 ± 0.04	0.20 ± 0.04
Ferritin (ug/L)	28.0 ± 12.6	33.0 ± 13.5	31.4 ± 9.4
Transferrin (g/L)	2.61± 0.30	2.59 ± 0.39	2.63 ± 0.37
Glucose (mmol/L)	4.55 ± 0.35	4.52 ± 0.39	4.57 ± 0.63
Insulin (uU/mL)	4.23 ± 2.92	3.72 ± 2.37	3.07 ± 3.22
Leptin (ng/mL)	4.45 ± 4.03	2.73 ± 2.03*	3.13 ± 1.77
Ghrelin (pmol/L)	385 ± 143	461 ± 135	378 ± 204
IGF-1 (nmol/L)	19.7 ± 7.3	18.0 ± 7.3	31.1 ± 18.0*
IGFBP-3 (ug/mL)	4.07 ± 1.08	4.02 ± 0.87	5.82 ± 2.75*
P1NP (ng/mL)	554 ± 176	458 ± 93*	620 ± 264
C telopeptides (pg/mL)	0.545 ± 0.205	0.504 ± 0.169	0.886 ± 0.518*
Osteocalcin (ng/mL)	50.9 ± 21.9	54.4 ± 13.7	80.9 ± 22.2**
Parathyroid hormone (pmol/L)	1.40 ± 0.96	1.22 ± 0.62	1.99 ± 1.22
Vitamin D (nmol/L)	67.6 ± 23.0	71.6 ± 21.4	69.1 ± 15.8

Mean ± standard deviation shown.

* p<0.05 **p<0.01 compared to baseline, paired *t*-test.

P1NP: propeptide of type I collagen; IGF-1: insulin-like growth factor 1; IGFBP-3: insulin-like growth factor binding protein 3; Vitamin D: 25-hydroxy vitamin D;

There were significant reductions in P1NP, leptin and albumin in the first 3 months. Over 3 years there were significant increases in IGF-1, IGFBP-3, and C telopeptides and osteocalcin.

We found no significant differences between the effects of dexamphetamine and methylphenidate on any of the parameters measured.

### 0–3 month changes predicting subsequent changes in body composition variables

Changes in the concentrations of IGF-1 and IGFBP-3 in the first 3 months were significant predictors of the changes in lean tissue from 6 months to 3 years (r=0.72, p=0.028 and r=0.74, p=0.022 respectively); in addition the changes in IGFBP-3 also correlated inversely with the subsequent changes in fat mass (r=−0.95, p<0.001). The changes in osteocalcin in the first 3 months correlated with the changes in BMD from 6 months to 3 years (r=0.77, p=0.026).

## Discussion

To our knowledge this is the first study to utilize DXA for determining the actual and relative losses of tissue when children start treatment with stimulant medication and to relate these to biochemical changes. The serum concentrations of leptin were decreased after starting treatment, correlating with the significant loss of fat mass. There was an early relative decrease in bone mass associated with a reduction in bone formation, with a significant recovery after three years of treatment associated with a recovery in cell coupling and bone turnover. In contrast, serum concentrations of vitamin D and PTH remained stable. At baseline the ADHD subjects had greater central adiposity than the DXA controls. The proportion of central fat declined on treatment, in parallel with progressive reductions in weight and BMI Z-scores. Over 3 years the lean tissue including bone increased more slowly than would be expected for growth in height.

The main strength of this study is in the combination of body composition analysis with biochemical parameters and regular, careful monitoring of height and weight. This methodology allowed us to relate the changes in growth parameters and body composition with our characterization of changes in serum metabolic markers, calciotropic hormones and bone turnover markers. However, the study was limited by the small number of timepoints for evaluating longitudinal changes. Another limitation of the study is the relatively small size of the cohort, which may have been the reason that the reductions in IGF-1, IGFBP-3 and CTX at 3 months did not reach statistical significance. This study is also limited by the lack of longitudinal control data. Although there were no untreated children with ADHD for longitudinal comparison, we were able to show significant reductions in the rates of increase in height and weight on starting treatment. This is consistent with stimulant associated reductions in the growth rates [4]. Another limitation is that the subjects and controls were not analysed on the same DXA machine. However, we principally used the controls’ data as a reference for longitudinal analysis of changes within subjects. For this, minor differences between machines would be far less important than they would be for the direct comparisons of the subjects with the controls. Using linear modeling controlling for age meant that all the controls could be compared with the subjects at every time point, maximising statistical power. It was not possible to evaluate the changes in relation to physical maturation as only one girl and one boy had entered puberty at the 3 year review. When we included stage of puberty in the analysis there was no significant effect, which we attribute to insufficient numbers.

There has been one previous cross sectional study investigating BMD and bone turnover in 10 boys who had been taking methylphenidate for 1–2 years compared to 10 healthy boys matched for age, height and weight [10]. The boys had had no changes in growth percentiles on treatment. This study reported no significant differences in BMD, serum bone-specific alkaline phosphatase or in urinary deoxypyridinoline in the study group as compared to the controls. In contrast, using more specific methods to quantify bone turnover, we have identified a significant decrease in bone formation (P1NP) during the early phase of the treatment and a “catch up” phenomenon at year 3 for both bone formation and bone resorption.

Our finding of greater weight and BMI Z-scores in the subjects than that of the DXA controls is consistent with the findings of Holtkamp et al. who found a higher rate of obesity in untreated children with ADHD than community controls [11]. We were also able to identify a higher proportion of central fat and a higher baseline BMD. As children approach puberty their total fat increases, together with their proportion of central fat [12]. However, in the subjects the proportion of central fat decreased with time. Central body fat distribution in children and adolescents is associated with increased cardiovascular and metabolic risk factors [13]. The relative improvement in fat distribution experienced by the subjects in this study contrasts with the pattern of increasing abdominal fat in relation to age among adolescents within their local community [12].

Our findings of initial preferential loss of central fat are consistent with findings from a systematic review of weight loss in adults that included 61 studies [14]. More weight loss over longer periods showed losses of central and peripheral fat in comparable proportions. The changes in body composition were not specific to the method employed for weight reduction; methods included exercise, diet, medication and surgery. Our study of weight loss on stimulant medication may therefore supply detail about the changes in body composition that might be anticipated if growing children lose weight.

## Conclusions

The effects of treatment in childhood on health in adult life are important. The present study shows that even relatively minor reductions in weight on stimulant medication can be associated not only with acute effects on fat and bone turnover but also with long-term changes in body composition. The improvement we observed in the proportion of central fat for height might reduce the long term cardiovascular and metabolic risk. However, any benefit would need to be evaluated in relation to the slower rate of increase in lean tissue including bone. Further study is required to determine the effects of these changes on adult health.

## Competing interests

The authors declare that they have no competing interests.

## Authors’ contributions

AP conceived the study, recruited and enrolled the participants, was involved in data collection and study coordination, statistical analysis and interpretation and writing the paper. JB contributed to data collection and statistical analysis. TM and MH carried out biochemical analysis including the bone biomarkers. EM contributed to the data collection and study coordination. LAB contributed to the study design and data interpretation. GD contributed to the study design and data interpretation. All authors were involved in revision of the manuscript and approved the final version.
